# TPPP3 Overexpression Suppresses Nasopharyngeal Carcinoma Progression and Promotes Immune Microenvironment Remodeling Through HSPA8 Association

**DOI:** 10.3390/cancers18172851

**Published:** 2026-09-03

**Authors:** Shengwei Li, Jiejun Liao, Jiawei Yang, Yanfeng Han, Zixiao Lei, Zheng Yang

**Affiliations:** Department of Clinical Laboratory, The First Affiliated Hospital of Guangxi Medical University, Nanning 530021, China

**Keywords:** TPPP3, nasopharyngeal carcinoma, immune microenvironment, HSPA8

## Abstract

**Objectives:** Nasopharyngeal carcinoma (NPC) arises in an immune-suppressive milieu that frequently undermines treatment efficacy. TPPP3 has been implicated as a negative regulator of NPC aggressiveness, yet its relevance to immune modulation or chaperone networks remains poorly defined. We therefore sought to determine how TPPP3 shapes the NPC immune landscape and to identify its interacting protein partners. **Methods:** Public single-cell RNA-sequencing datasets from head and neck squamous cell carcinoma and nasopharyngeal carcinoma were analyzed using R. HK-1 and C666-1 cells stably overexpressing TPPP3 were established. These cells were used to construct humanized xenograft tumor models, with intratumoral immune cell infiltration evaluated by immunohistochemistry. In vitro, the same cells and their controls were indirectly co-cultured with peripheral blood mononuclear cells. Cellular lysates from TPPP3-overexpressing cells were subjected to immunoprecipitation–mass spectrometry and immunofluorescence staining, which identified HSPA8 as a TPPP3-interacting protein. Three groups—control, TPPP3-overexpressing, and TPPP3-overexpressing plus the HSPA8 inhibitor VER155008—were then compared in wound healing, colony formation, cell-cycle, and xenograft assays, with immunohistochemical staining for Ki67, TPPP3, and CD3 performed on tumor sections. **Results:** TPPP3 transcripts were barely detectable across most tumor cell subsets but showed preferential enrichment in NPC epithelial clusters. Enforced TPPP3 expression curtailed xenograft outgrowth while increasing intratumoral abundance of CD3^+^ T cells, CD8^+^ T cells, and CD11c^+^ dendritic cells. Pharmacological blockade of HSPA8 with VER155008 further enhanced TPPP3-driven suppression of migration, clonogenicity, and tumor expansion, and also altered cell-cycle progression while boosting CD3^+^ T-cell accumulation within grafts. **Conclusions:** These findings suggest a functional association between TPPP3 and HSPA8 that may contribute to tumor growth suppression and immune microenvironment remodeling in NPC. Pharmacological disruption of HSPA8-dependent proteostasis enhanced TPPP3-associated antitumor activity in both in vitro and in vivo models, indicating that this chaperone pathway represents a candidate mechanism worthy of further mechanistic investigation and therapeutic exploration.

## Simple Summary

Nasopharyngeal carcinoma is a head and neck cancer linked to Epstein–Barr virus infection. Some patients respond well to immunotherapy, but many do not, and the reasons for this difference remain unclear. In this study, we investigated a protein called TPPP3, which is present at low levels in nasopharyngeal carcinoma cells. We found that increasing TPPP3 in cancer cells slowed tumor growth in both cell models and mice with humanized immune systems. It also attracted more cancer-fighting immune cells, including T cells and dendritic cells, into the tumor. We further discovered that TPPP3 physically interacts with another protein, HSPA8, which helps maintain protein balance within cells. When we blocked HSPA8 with a drug, the tumor-suppressing effects of TPPP3 became even stronger. These findings suggest that targeting the interaction between TPPP3 and HSPA8 could offer a new approach to improving immunotherapy outcomes for patients with nasopharyngeal carcinoma.

## 1. Introduction

Nasopharyngeal carcinoma (NPC) is an epithelial malignancy closely linked to Epstein–Barr virus (EBV) infection, characterized by significant geographical clustering and population-specific distribution [1]. EBV-encoded products facilitate the malignant transformation of epithelial cells by activating various signaling pathways, including NF-κB and PI3K/Akt, while concurrently inducing an immunosuppressive microenvironment [2,3]. Single-cell transcriptomic analyses have demonstrated that levels of CD8^+^ cytotoxic T cell exhaustion and the density of CD11c^+^ dendritic cell infiltration in NPC tissues are significantly associated with patient prognosis and responses to immunotherapy [4,5,6]. Dendritic cell dysfunction directly contributes to the immune “cold” tumor phenotype and represents a key mechanism limiting therapeutic efficacy [7]. In NPC, the functional state of immune infiltrates influences the efficacy of immunotherapy [8].

The tubulin polymerization-promoting protein family member 3 (TPPP3) plays a key role in regulating microtubule dynamics and maintaining cytoskeletal homeostasis [9]. In head and neck squamous cell carcinoma, the low expression level of TPPP3 is closely related to poor prognosis and reduced infiltration of anti-tumor immune cells [10]. In contrast, its upregulation exhibits pro-tumor functions in non-small cell lung cancer [11], glioblastoma [12], endometrial cancer [13], and breast cancer [14]. TPPP3 acts as a tumor suppressor in OSCC and NPC, with high expression predicting better outcomes [15,16]. Preliminary data from our studies show that TPPP3 is predominantly localized in NPC epithelial cells and positively correlates with the infiltration of CD8^+^ T cells and CD11c^+^ dendritic cells, suggesting a potential role in immune regulation, although the underlying mechanisms require further exploration.

Heat shock-associated protein 71-kDa (HSPA8, also known as HSC70) is a constitutively expressed molecular chaperone involved in protein folding, chaperone-mediated autophagy and antigen presentation [17]. In the inflammatory state and a variety of cancers, its expression is often abnormally regulated. Functionally, HSPA8 is involved in promoting the progression of tumors such as gastric cancer, hepatocellular carcinoma, and cholangiocarcinoma through a variety of different mechanisms [18,19,20]. It is worth noting that HSPA8 can directly bind to and stabilize MHC-II molecules, thereby regulating antigen presentation and adaptive immunity [21]. However, the direct interaction between TPPP3 and HSPA8 and the functional significance of this interaction in NPC have not been studied.

In summary, this study aims to clarify the tumor suppression effect of TPPP3 in NPC, verify the functional importance of TPPP3-HSPA8 association, and provide a theoretical basis for improving the selection and design of targeted interventions for immunotherapy patients.

## 2. Materials and Methods

### 2.1. Bioinformatics Analysis and Public Data Mining

Publicly available transcriptomic data were obtained from the Gene Expression Omnibus (GEO). The HNSCC single-cell dataset GSE103322 was used to examine TPPP3 distribution across malignant and non-malignant cell populations, and the NPC dataset GSE120926 was employed to characterize TPPP3 expression across epithelial, immune, and stromal compartments. All analyses were performed in R (version 4.5.0) using GEOquery (version 4.5.0) for data retrieval and Seurat (version 4.5.0) for single-cell data processing, clustering, and visualization. Bulk data were processed with GEOquery, followed by differential expression analysis using limma (version 4.5.0) or DESeq2 (version 4.5.0) with Benjamini–Hochberg correction. For single-cell data, quality control, normalization, scaling, and principal-component analyses were performed, followed by graph-based clustering and visualization via t-SNE and UMAP. Cell identities were assigned using published annotations and canonical marker genes.

### 2.2. Cell Lines and Establishment of TPPP3-Overexpressing Stable Cells

HK-1 and C666-1 cells were provided by the Otorhinolaryngology Laboratory of Guangxi Medical University (Nanning, China). Cells were cultured in RPMI-1640 with 10% FBS and 1% penicillin-streptomycin at 37 °C in 5% CO_2_. Lentiviral vectors encoding full-length TPPP3 or empty vector (both with GFP and puromycin markers) were obtained fromGenechem (Shanghai, China). Cells at 30–50% confluence were transduced at a multiplicity of infection (MOI) of 30 with 8 μg/mL Polybrene. After 12 h, the medium was replaced. Transduction efficiency was assessed by GFP fluorescence at 72 h. Stable cells were selected with puromycin (2 μg/mL for HK-1; 3 μg/mL for C666-1). All lentiviral procedures were performed under biosafety level 2 (BSL-2) conditions.

### 2.3. RNA Extraction and Quantitative Real-Time PCR (RT-qPCR)

Total RNA was extracted from HK-1 and C666-1 cells using RNAiso Plus reagent (TaKaRa, Shiga, Japan) and quantified using a NanoDrop 2000 spectrophotometer (Thermo Fisher Scientific, Waltham, MA, USA). cDNA synthesis was performed using a PrimeScript RT kit (TaKaRa, Shiga, Japan) containing a gDNA scavenger. The qPCR reaction was performed on the QuantStudio 7 Flex system (Applied Biosystems, Waltham, MA, USA) using TB Green Premix Ex Taq II (TaKaRa, Shiga, Japan) with GAPDH as an internal reference. The relative expression level was calculated by the 2^−^^ΔΔCt^ method. The primer sequences are as follows: GAPDH (forward 5′-TGATGGGTCACGTACCACGAGGAG-3′, reverse 5′-AGTGATGGCATGACTGTGGGGGGGG-3′) and TPPP3 (forward 5′-AAGTCTGCTCGGTCGATCAAC-3′, reverse 5′-GAGCCCGTATCTGCTGG-3′).

### 2.4. Western Blotting

HK-1 and C666-1 cells were lysed on ice with RIPA buffer, and protein concentration was measured. The same number of proteins were separated by SDS-PAGE and transferred to the membrane, and then immunostained with primary antibodies against TPPP3 (1:100, Proteintech, Wuhan, China), FLAG (1:2000, Abcam, Cambridge, UK), HSPA8 (1:1000, Proteintech, Wuhan, China), GAPDH (1:10,000, Abcam, Cambridge, UK), and β-tubulin (1:100,000, HUABIO, Huangzhou, China), followed by the addition of universal goat anti-rabbit secondary antibody (Proteintech, Wuhan, China). The bands were visualized by enhanced chemiluminescence (ECL) and quantitatively analyzed by ImageJ (version 1.53) [22].

### 2.5. Co-Culture Assay

PBMCs were isolated from healthy donors by Ficoll–Paque density gradient centrifugation. Prior to co-culture, PBMCs were activated with PHA-M. For indirect co-culture, TPPP3-overexpressing or control NPC cells (HK-1 or C666-1) were seeded in the upper chambers of 24-well Transwell plates, and activated PBMCs were placed in the lower chambers at a tumor-to-PBMC ratio of 1:10–1:20. After 24–48 h of co-culture, PBMCs were harvested from the lower chamber and analyzed by flow cytometry.

### 2.6. Flow Cytometry

Immune cell staining: PBMCs were washed twice with cold PBS (300–400 g, 5 min), then stained with anti-CD3 and anti-CD8 antibodies (BioLegend, San Diego, CA, USA) at 4 °C for 30 min in the dark. After a final wash, cells were acquired on a flow cytometer (BD Biosciences, Franklin Lakes, NJ, USA). Single-staining and FMO controls were used for gating. Data analysis excluded debris and doublets via FSC/SSC, followed by gating on CD3^+^ T cells and then CD8^+^ subpopulation.

Cell cycle analysis: Cells in the logarithmic growth phase were collected, fixed, stained with PI-RNase, and analyzed on the same flow cytometer (BD Biosciences) to determine the percentages of cells in G0/G1, S, and G2/M phases.

### 2.7. Co-Immunoprecipitation Coupled with Mass Spectrometry (IP-MS)

Cells expressing FLAG-TPPP3 or empty vector were lysed in ice-cold IP lysis buffer with protease inhibitors. A portion of lysate was saved as input. The remaining lysate was incubated with FLAG antibody pre-bound to protein A/G magnetic beads. After washing, bound proteins were eluted with FLAG peptide. The eluate was subjected to in-gel digestion with trypsin overnight at 37 °C following reduction and alkylation. Resulting peptides were desalted on C18 StageTips, redissolved in 0.1% formic acid, separated by nano-liquid chromatography, and analyzed on a Q-Exactive HF-X mass spectrometer (Thermo Fisher Scientific, Waltham, MA, USA) in data-dependent acquisition (DDA) mode.

### 2.8. Immunofluorescence Analysis

For immunofluorescence staining, the cells were seeded on a slide, fixed with 4% glutaraldehyde for 15 min, infiltrated with 0.1% Triton X-100 for 20 min, and blocked with normal goat serum at room temperature for 10 min. The slides were then incubated overnight at 4 °C using specific primary antibodies against TPPP3 (Proteintech, 15057-1-AP, 1:400) and HSPA8/HSC70 (Proteintech, 10654-1-AP, 1:400). After washing, HRP-labeled secondary antibody was added at room temperature for 30 min, and then TSA fluorescence imaging was performed (TYR570 and TYR690, 5 min each). The nucleus was reverse-stained with DAPI, and the slide was sealed with anti-fading gel medium. The images were collected by fluorescence microscope.

### 2.9. Animal Experiments

All experiments were performed on female huHSC-NCG mice, weighing 24–26 g, purchased from GemPharmatech (Nanjing, China), for 15 weeks, under adaptive feeding for one week, and mice were fed under SPF conditions throughout the experiment. Two groups of independent subcutaneous xenotransplantation experiments were performed. In experiment 1, 1 × 10^7^ HK-1 or C666-1 cells stably expressing TPPP3 (OE group) or empty vector (NC group) were injected into mice and divided into an NC group and an OE group without drug treatment. The mice were euthanized 2 weeks after transplantation. In experiment 2, HK-1 or C666-1 cells stably expressing TPPP3 (OE group) or empty vector (NC group) were also injected, and divided into an NC group, an OE group, and an OE+VER155008 group. On the second day of transplantation, 25 mg/kg VER155008 was intraperitoneally injected into the OE+VER155008 group, and 2% DMSO in normal saline was used as the solvent control in the NC group and the OE group. The tumor size was recorded every 3 days in both groups. At the end of the experiment (day 16 of experiment 2), the tumor tissue was removed, weighed, photographed, and fixed for histological analysis. All operations were approved by the Ethics Committee of Guangxi Medical University.

### 2.10. Immunohistochemistry (IHC)

Excised tumors were fixed in 4% paraformaldehyde, paraffin-embedded, and sectioned at 4 μm. After deparaffinization, rehydration, and antigen retrieval, sections were blocked for endogenous peroxidase and non-specific binding, then incubated overnight at 4 °C with primary antibodies against TPPP3, CD3, CD8, CD11c, and Ki-67 (Proteintech, 1:200). Following washing, sections were incubated with HRP-conjugated secondary antibody for 1 h at room temperature, visualized with DAB, counterstained with hematoxylin, and imaged under a light microscope. At least 3 sections from different mice per group and 5 randomly selected fields per section were quantified.

### 2.11. Colony Formation Assay

Exponentially growing cells were plated into 6-well plates (500–1000 cells/well) with three groups: NC, OE (TPPP3 overexpression), and OE+VER155008. NC and OE were cultured in normal medium; OE+VER155008 was cultured in medium supplemented with 5 μM VER155008 (Supplementary Figure S1). After 10–14 days (medium changed every 3–4 days), cells were fixed with 4% paraformaldehyde, stained with 0.1% crystal violet, and air-dried. Colonies (≥50 cells) were counted.

### 2.12. Detection of Wound Healing

Exponentially growing cells were seeded in 6-well plates. After 24 h, a straight-line scratch was made in each well using a sterile 200 μL pipette tip, and then the exfoliated cells were gently washed with PBS to remove the exfoliated cells. Subsequently, the medium was replaced with serum-free medium and incubated for 24 h. Under an inverted microscope, images of the same area were taken at 0 h and 24 h, and the wound area was measured using ImageJ software.

### 2.13. Statistical Analysis

GraphPad Prism 9.0 was used for data analysis and graphing. Continuous variables were tested for normality and variance homogeneity. Normally distributed data with equal variances are presented as mean ± SD; two-group comparisons used independent *t*-tests, and multiple groups were analyzed by one-way ANOVA with post-hoc tests. Non-normally distributed data were analyzed using non-parametric tests. Multiple comparisons were adjusted by false discovery rate (FDR). All experiments were performed with at least three independent biological replicates. *p* < 0.05 was considered statistically significant.

## 3. Results

### 3.1. TPPP3 Expression Patterns in HNSCC and NPC Based on GEO Single-Cell Data

**Figure 1:**
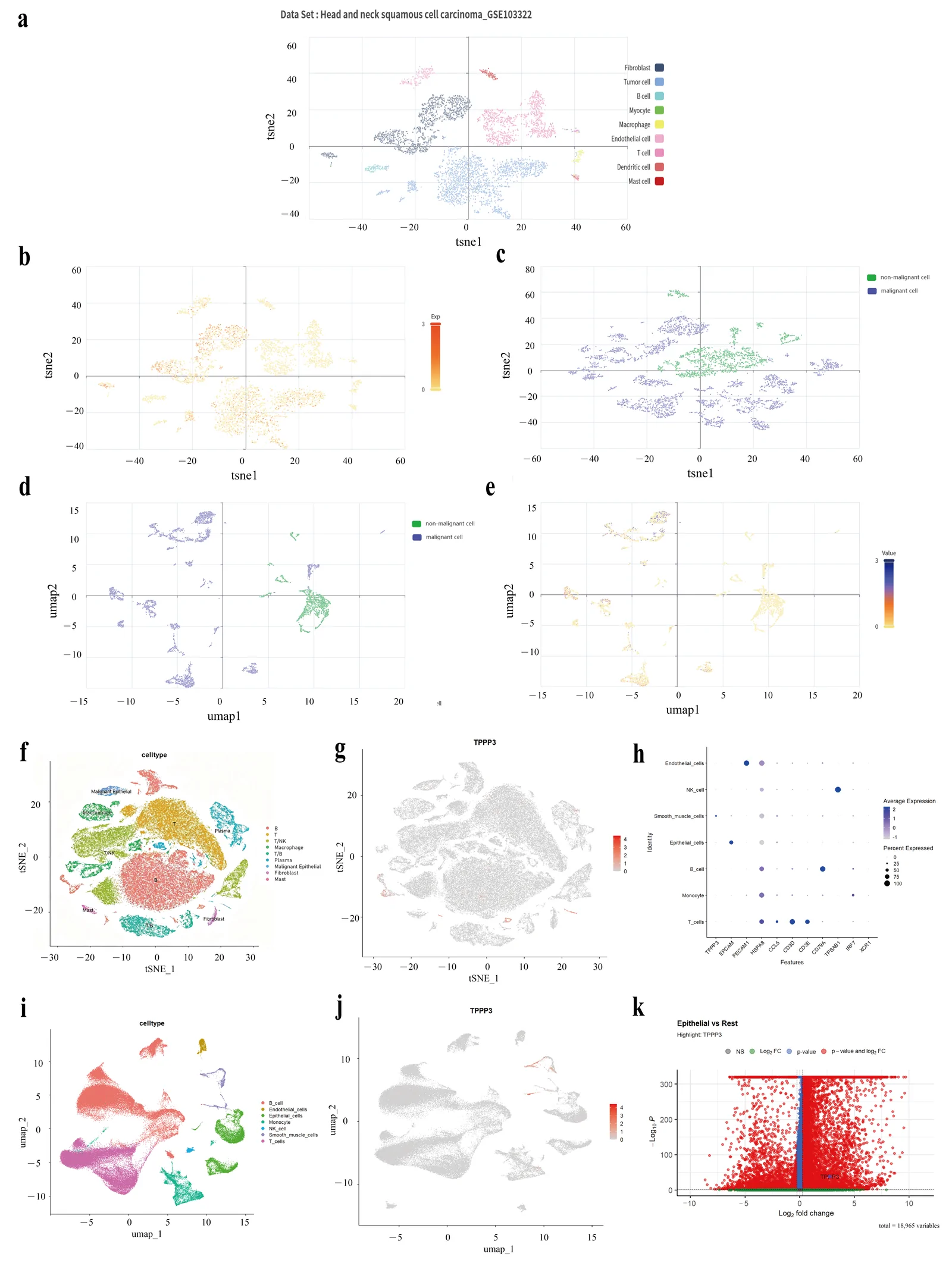
TPPP3 expression in HNSCC and NPC based on public single-cell datasets. (**a**) t-SNE map of annotated HNSCC cell populations. (**b**) TPPP3 expression on the HNSCC t-SNE map. (**c**) t-SNE distribution of malignant and non-malignant HNSCC cells. (**d**) UMAP map of malignant and non-malignant HNSCC cells. (**e**) TPPP3 expression on the HNSCC UMAP map. (**f**) t-SNE map of annotated NPC cell populations. (**g**) TPPP3 expression in NPC (t-SNE). (**h**) Bubble plot showing TPPP3 and marker-gene expression across NPC populations; color denotes average expression and dot size denotes the proportion of expressing cells. (**i**) UMAP map of annotated NPC cell populations. (**j**) TPPP3 expression in NPC (UMAP). (**k**) Volcano plot showing TPPP3 expression comparing NPC epithelial cells with other annotated cell populations.

To assess TPPP3 expression in the head and neck squamous cell carcinoma (HNSCC) microenvironment, single-cell transcriptomic data from GSE103322 were analyzed. t-SNE and UMAP revealed uniformly low TPPP3 expression across all cell populations, with sporadic signals observed in malignant cells and little to no signal in non-malignant populations (Figure 1a–e). Malignant cells formed multiple discrete clusters, indicating substantial intratumoral transcriptional heterogeneity, whereas non-malignant cells aggregated in the central region (Figure 1c). Analysis of GSE120926 similarly showed weak TPPP3 signals across most annotated NPC cell populations, including malignant epithelial cells, fibroblasts, endothelial cells, B cells, T cells, and macrophages (Figure 1f–j). Dot-plot analysis confirmed low TPPP3 average expression and detection rates across all cell populations (Figure 1h). Nevertheless, differential expression analysis revealed relative enrichment of TPPP3 in epithelial cells compared with other populations (avg log2FC = 2.75, *p* < 0.0001) (Figure 1k). Thus, TPPP3 was broadly low in absolute expression but showed a relative epithelial preference in NPC, supporting the subsequent use of epithelial NPC cell models.

### 3.2. Construction and Validation of Lentivirus-Mediated TPPP3-Overexpressing Cell Models

**Figure 2:**
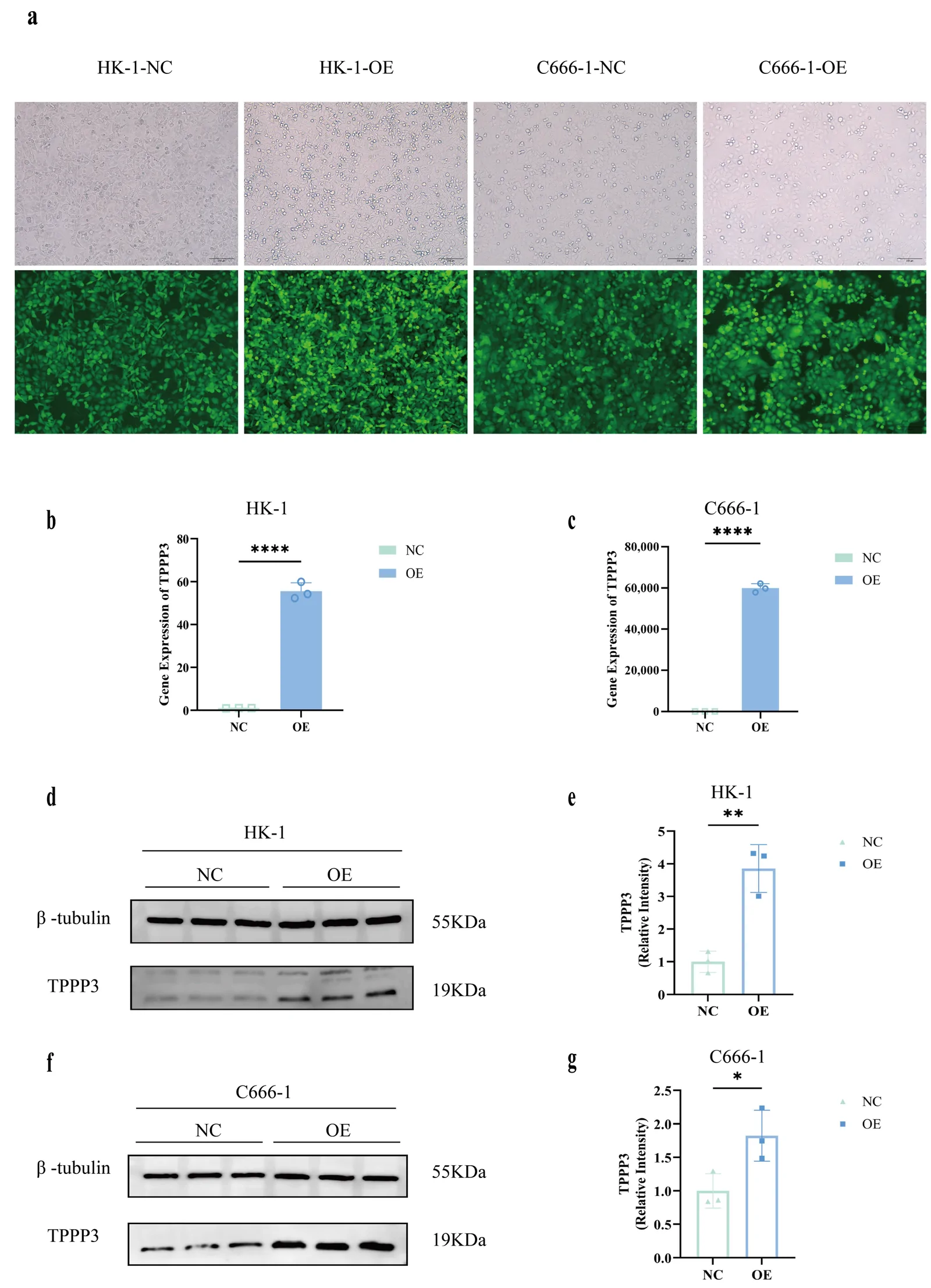
Validation of stable TPPP3 overexpression in HK-1 and C666-1 cells. (**a**) GFP fluorescence images after lentiviral transduction. (**b**,**c**) RT-qPCR analysis of TPPP3 mRNA in HK-1 and C666-1 cells, respectively. Western blot and densitometric quantification of TPPP3 protein levels in HK-1 (**d**,**e**) and C666-1 (**f**,**g**) cells. * *p* < 0.05, ** *p* < 0.01,**** *p* < 0.0001. The whole blots (uncropped blots) are shown in the Supplementary Materils File.

Given the low endogenous expression level of TPPP3 in nasopharyngeal carcinoma (NPC), an overexpression strategy was adopted. HK-1 and C666-1 cells were transduced with GFP-expressing lentiviral vectors carrying TPPP3 or the empty control. A high proportion of GFP-positive cells was observed 72 h after transduction, and stable cell populations were obtained after puromycin selection (Figure 2a). RT-qPCR demonstrated significantly higher TPPP3 mRNA abundance in OE cells than in NC cells in both models (Figure 2b,c). Western blotting confirmed increased TPPP3 protein expression in HK-1 OE cells (Figure 2d,e) and C666-1 OE cells (Figure 2f,g). These results verified stable TPPP3 overexpression at both the transcript and protein levels.

### 3.3. TPPP3 Overexpression Suppresses Xenograft Growth and Remodels Immune-Cell Infiltration

#### 3.3.1. In Vivo Suppression of Tumor Growth and Enhancement of Immune Infiltration

**Figure 3:**
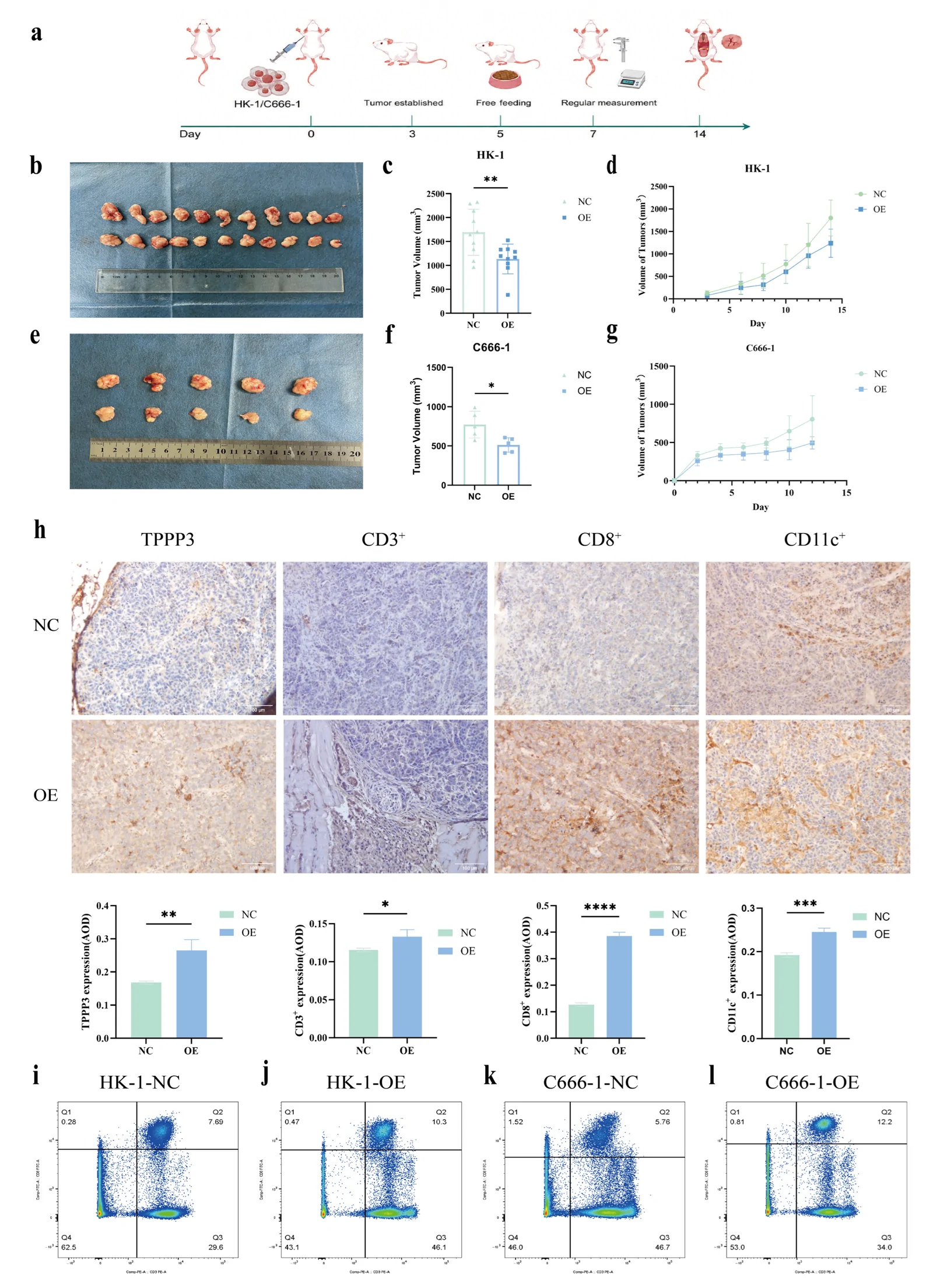
TPPP3 overexpression inhibited the growth of xenograft tumors and enhanced the infiltration of immune cells in tumors. (**a**) The schematic diagram of huHSC-NCG xenotransplantation experiment. (**b**) Representative images of HK-1 tumor xenografts. (**c**) Quantitative results of endpoint tumor volume in HK-1 model. (**d**) Tumor growth curves of HK-1 xenografts. (**e**) Representative images of C666-1 tumor xenografts. (**f**) Quantitative results of endpoint tumor volume in C666-1 model. (**g**) Tumor growth curves of C666-1 xenografts. (**h**) Immunohistochemical staining and corresponding quantitative analysis of CD3^+^, CD8^+^, and CD11c^+^ cells in tumor sections. (**i**–**l**) PBMC was co-cultured with HK-1 or C666-1 cells, and CD3^+^ and CD8^+^ T cells were analyzed by flow cytometry. The data were expressed as mean ± standard deviation. * *p* < 0.05, ** *p* < 0.01, **** *p* < 0.0001.

To determine the functional role of TPPP3 in NPC tumorigenesis and immune modulation, HK-1 and C666-1 xenograft models were generated in huHSC-NCG mice with TPPP3-overexpressing (OE) or control (NC) cells (Figure 3a). At the experimental endpoint, tumors derived from TPPP3-overexpressing cells were smaller than the corresponding control tumors in both models (Figure 3b–g). In the HK-1 model, mean tumor volume was 1388 ± 335.6 mm^3^ in the NC group and 751.7 ± 220.9 mm^3^ in the OE group (Figure 3c). In the C666-1 model, mean tumor volume was 772.9 ± 170.6 mm^3^ in the NC group and 512.1 ± 91.67 mm^3^ in the OE group (Figure 3e).

Immunohistochemical analysis showed higher densities of CD3^+^ T cells, CD8^+^ T cells, and CD11c^+^ dendritic cells in TPPP3 OE tumors than in NC tumors (Figure 3h). Thus, TPPP3 overexpression was associated with reduced xenograft growth and increased infiltration of the measured T-cell and dendritic-cell populations.

#### 3.3.2. In Vitro Assessment of T Cell Activation by Transwell Co-Culture

To determine whether TPPP3 overexpression in NPC cells directly affects immune cells via soluble factors, an indirect Transwell co-culture system was established using human PBMCs from multiple donors and HK-1 or C666-1 cells stably expressing TPPP3-OE or control vector; the proportion of CD3^+^CD8^+^ T cells was then analyzed by flow cytometry (Figure 3i–l). A modest upward trend in CD3^+^CD8^+^ T-cell proportion was observed in PBMCs from some donors after co-culture with OE cells; however, due to differences among donors, the pooled comparison did not reach statistical significance in either the HK-1 or C666-1 system. These data suggest that the response under this simplified ex vivo condition is heterogeneous and may depend on individual donor characteristics and additional tumor-microenvironmental components.

### 3.4. HSPA8 Was Identified as a Candidate Protein for TPPP3-Related Proteins

**Figure 4:**
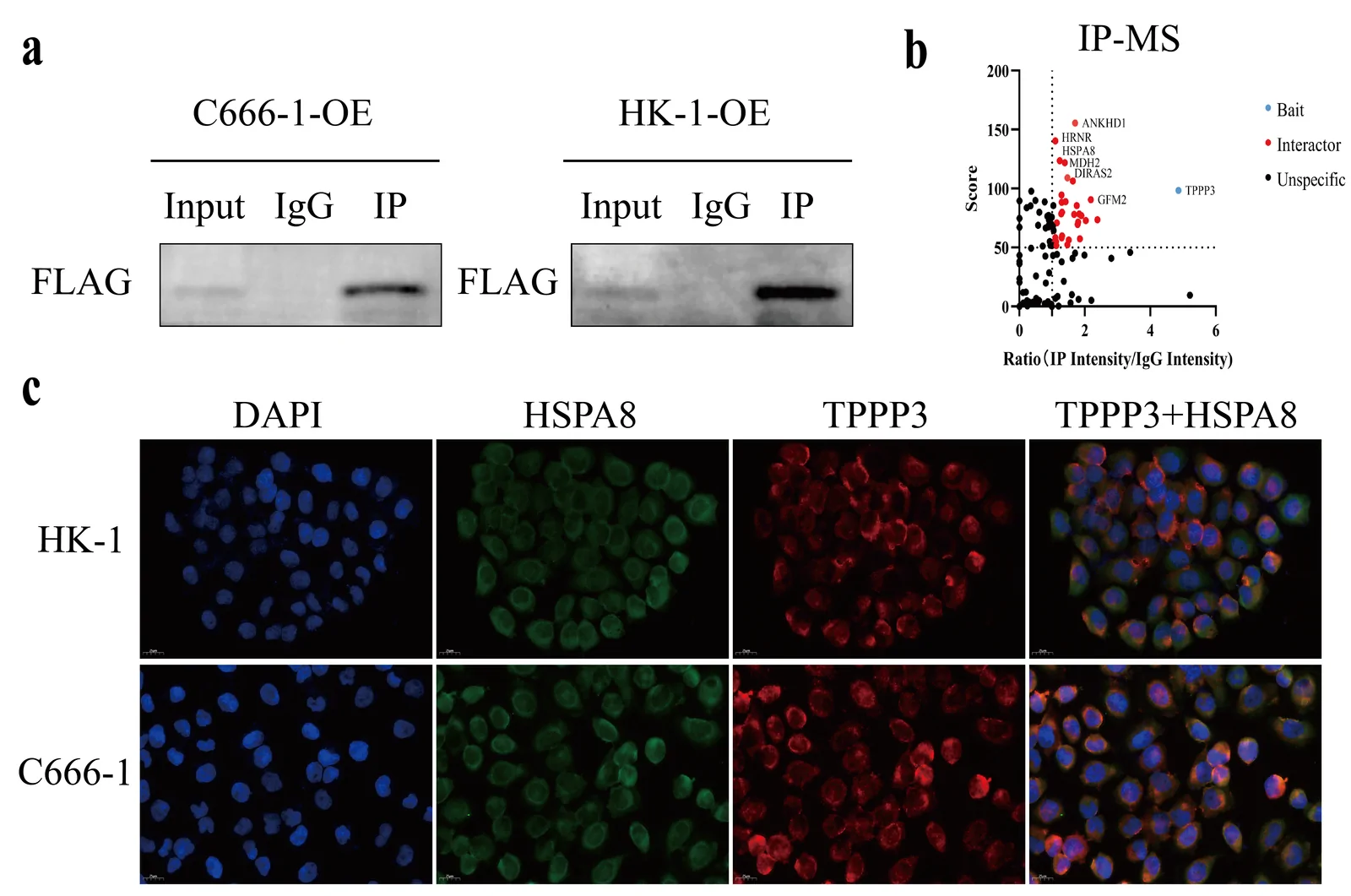
HSPA8 was identified as a TPPP3-related candidate protein by IP-MS. (**a**) Western blot analysis of FLAG-TPPP3 after anti-FLAG immunoprecipitation. (**b**) Enrichment map and identification score of candidate TPPP3 interacting proteins in IP-MS analysis. (**c**) Immunofluorescence images showing nuclei (blue), HSPA8 (green), TPPP3 (red) and merged channels. The whole blots (uncropped blots) are shown in the Supplementary Materils File.

The protein lysates collected from HK-1 and C666-1 cells stably overexpressing FLAG-TPPP3 were subjected to FLAG-based immunoprecipitation analysis. FLAG-TPPP3 was efficiently recovered in anti-FLAG immunoprecipitation, which confirmed the successful enrichment of bait proteins (Figure 4a). Mass spectrometry analysis of immunoprecipitates showed that HSPA8 was continuously enriched in both cell lines, and the identification scores were 123.64 and 133.71, respectively (Figure 4b). The complete list of candidate proteins identified by IP-MS, along with the detailed identification statistics and peptide identification tables, is provided in Supplementary Tables S1–S3. In view of the repeatable enrichment results in both models, HSPA8 was selected as a candidate for further evaluation of TPPP3-related proteins. Immunofluorescence analysis further showed that both proteins were localized in the cytoplasm (Figure 4c).

### 3.5. HSPA8 Inhibition Potentiates TPPP3-Associated Antitumor Phenotypes in NPC Cells

**Figure 5:**
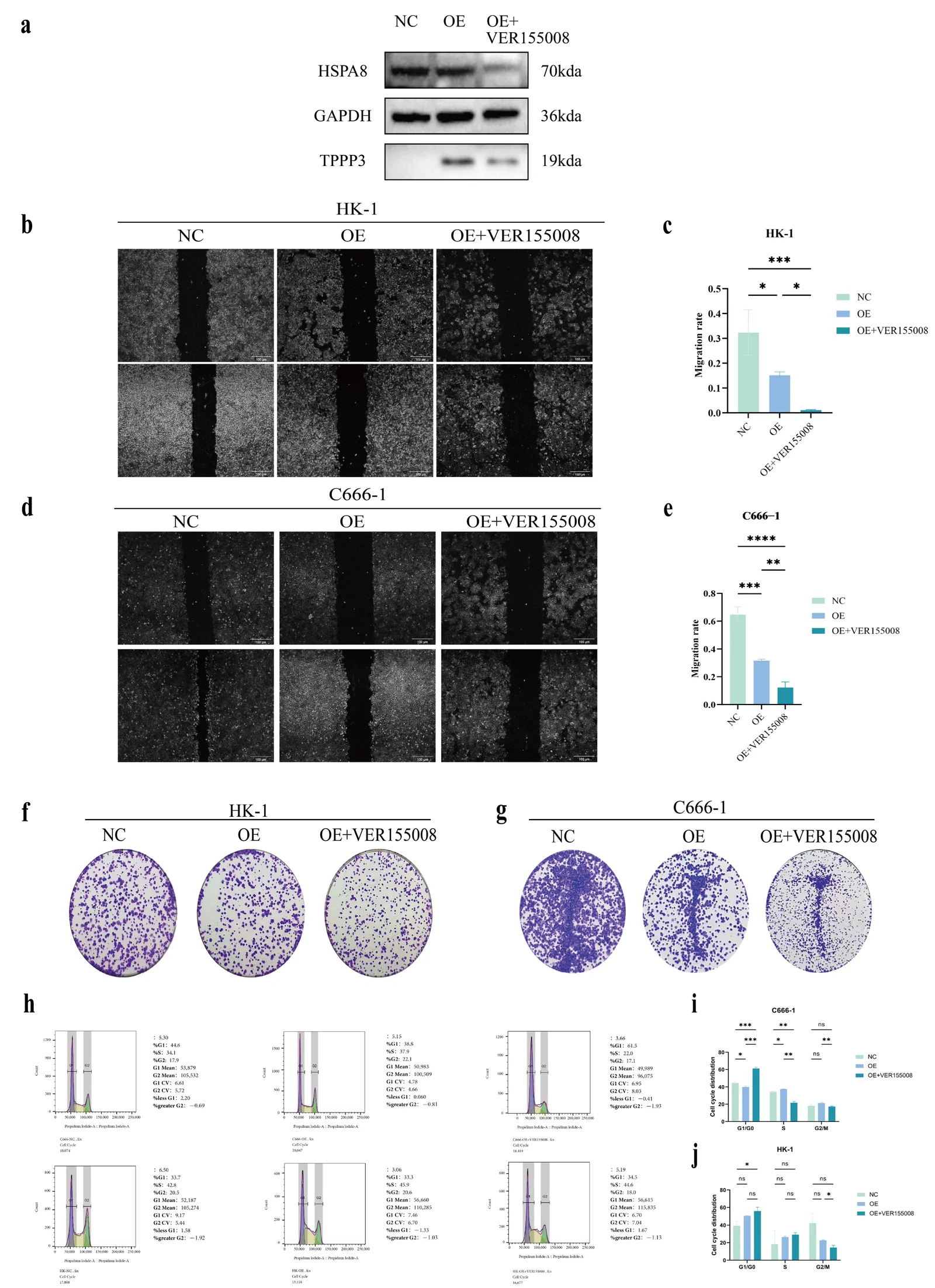
HSPA8 inhibition enhances antitumor phenotypes in TPPP3-overexpressing cells. (**a**) Immunoblotting of HSPA8 and TPPP3 in NC, OE, and OE+VER155008 cells. (**b**,**c**) Representative wound-healing images and quantification in HK-1 cells at 0 and 24 h. (**d**,**e**) Representative wound-healing images and quantification in C666-1 cells at 0 and 24 h. (**f**,**g**) Colony formation assay in HK-1 and C666-1 cells. (**h**) Flow-cytometric profiles of cell-cycle distribution in C666-1 cells (**upper**) and HK-1 cells (**lower**). (**i**,**j**) Statistical analysis of cell cycle phases in C666-1 cells and HK-1 cells. * *p* < 0.05, ** *p* < 0.01, *** *p* < 0.001, **** *p* < 0.0001. The whole blots (uncropped blots) are shown in the Supplementary Materils File.

To test this, the HSPA8/HSP70 inhibitor VER155008 was applied to TPPP3-overexpressing HK-1 and C666-1 cells. VER155008 treatment compromised HSPA8 stability and reduced its protein abundance, accompanied by a modest decrease in TPPP3 expression (Figure 5a).

Wound-healing assays revealed that OE cells exhibited significantly lower wound closure rates than NC cells, and this effect was further enhanced upon VER155008 treatment (Figure 5b–e). Similarly, colony formation assays showed that both the number and size of colonies were markedly reduced in OE cells and further decreased after VER155008 addition (Figure 5f,g).

Flow cytometric analysis revealed distinct cell-cycle responses to TPPP3 overexpression and VER155008 treatment in HK-1 and C666-1 cells (Figure 5h–j). In HK-1 cells, TPPP3 overexpression alone did not significantly alter cell-cycle distribution. However, combined treatment with VER155008 led to marked G0/G1 arrest and a reduction in G2/M proportions, while the S-phase fraction remained unchanged. Conversely, in C666-1 cells, TPPP3 overexpression resulted in a modest increase in G0/G1 and a decrease in S-phase populations. The addition of VER155008 further augmented G0/G1 accumulation and inhibited S-phase entry. These findings indicate that HSPA8 inhibition enhances the TPPP3-mediated G0/G1 cell-cycle blockade in a context- and model-specific manner.

### 3.6. HSPA8 Inhibition Combined with TPPP3 Overexpression Suppresses Xenograft Growth and Potentiates Intratumoral CD3^+^ T-Cell Infiltration

**Figure 6:**
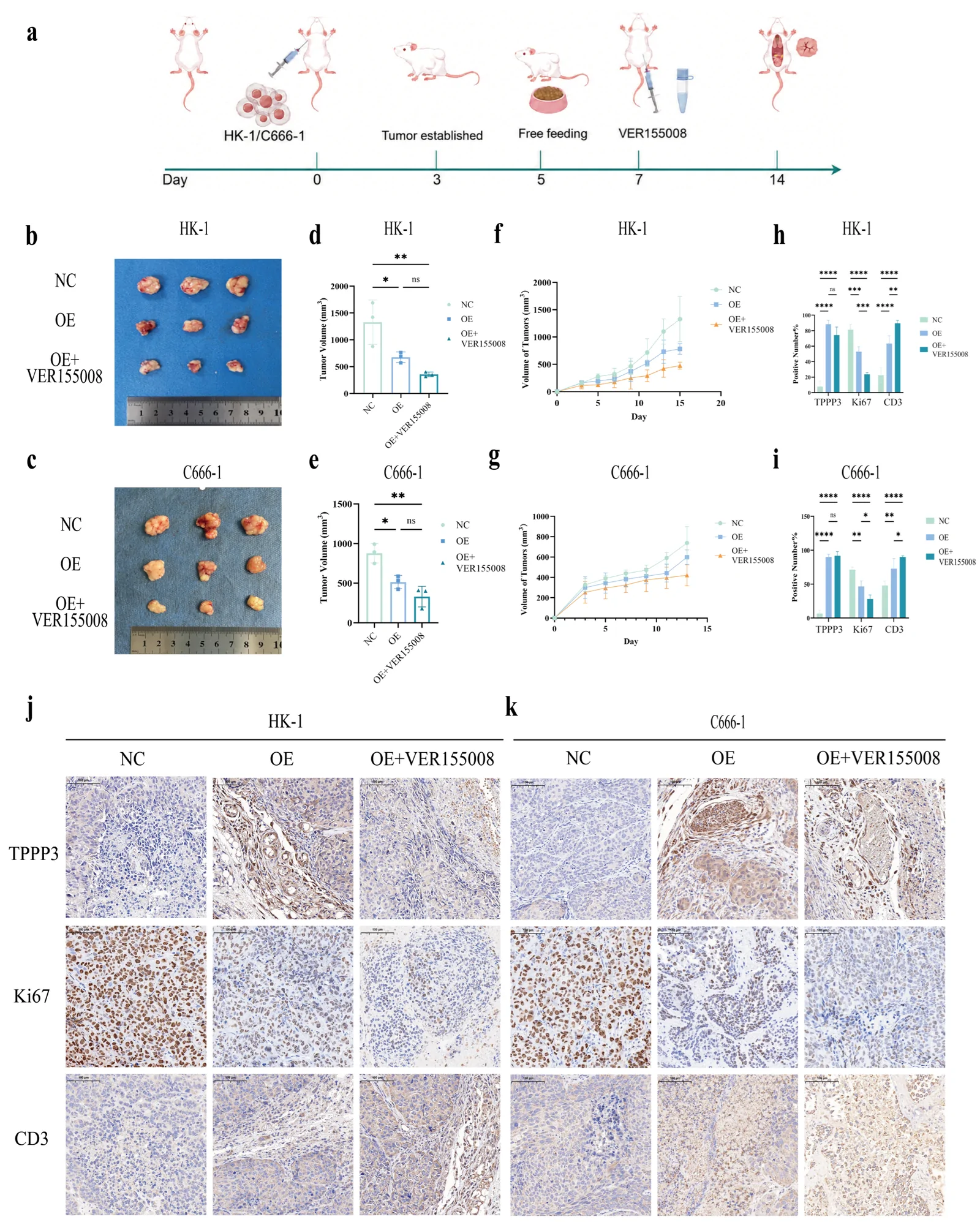
HSPA8 inhibition enhances tumor suppression in TPPP3-overexpressing xenografts. (**a**) Schematic illustration of the in vivo experimental protocol. (**b**,**c**) Representative photographs of resected tumors from each group. (**d**,**e**) Statistical bar graphs of tumor volumes. (**f**,**g**) Tumor-growth curves of HK-1 and C666-1 xenografts. (**h**–**k**) Immunohistochemical staining and quantitative analysis of TPPP3, Ki-67, and CD3 expression in tumor tissues. * *p* < 0.05, ** *p* < 0.01, *** *p* < 0.001, **** *p* < 0.0001.

To validate the enhanced antitumor effects observed in vitro, huHSC-NCG mice bearing HK-1 or C666-1 xenografts were treated with VER155008 (Figure 6a). Tumor volume measurements revealed that OE tumors were significantly smaller than NC tumors, with further reductions upon VER155008 treatment in both models (Figure 6b–g).

Immunohistochemical analysis of tumor sections showed weak, focal TPPP3 staining in NC tumors, whereas OE tumors exhibited stronger and more diffuse staining; the TPPP3 signal was slightly reduced in the OE+VER155008 group compared with OE alone, but remained above NC levels (Figure 6j,k). Ki-67 staining was markedly decreased in both OE and OE+VER155008 groups relative to NC tumors. Conversely, CD3^+^ T-cell infiltration was sparse in NC tumors, increased in OE tumors, and further enhanced upon VER155008 treatment. Quantitative analyses confirmed significant differences among the three groups for all three markers (Figure 6h,i).

## 4. Discussion

Nasopharyngeal carcinoma (NPC) is an Epstein–Barr virus-associated epithelial malignancy characterized by a highly immunosuppressive tumor microenvironment [23,24]. While induction chemotherapy combined with concurrent chemoradiotherapy is the standard approach for locally advanced disease [25], the use of PD-1 inhibitors alongside gemcitabine-cisplatin has led to improved outcomes in recurrent/metastatic NPC [26]. Nevertheless, challenges such as distant metastasis, local recurrence, and heterogeneous responses to immunotherapy persist [27].

The status of the tumor microenvironment critically influences the efficacy of immunotherapy, as EBV-mediated immune dysregulation, T-cell exhaustion, and chemokine imbalances collectively contribute to immune evasion in NPC [28]. TPPP3, a microtubule polymerization-promoting protein, primarily participates in cytoskeletal remodeling and microtubule homeostasis [9]. Notably, analysis of the GSE12452 and GSE53819 datasets has demonstrated that TPPP3 is expressed at significantly lower levels in NPC tissues than in normal nasopharyngeal tissues [10]. Although TPPP3 is generally expressed at low levels in NPC, its preferential enrichment in epithelial cells leads us to hypothesize that it plays a crucial role as a key hub in coordinating nasopharyngeal epithelial homeostasis and tumor immune activation.

**Figure 7:**
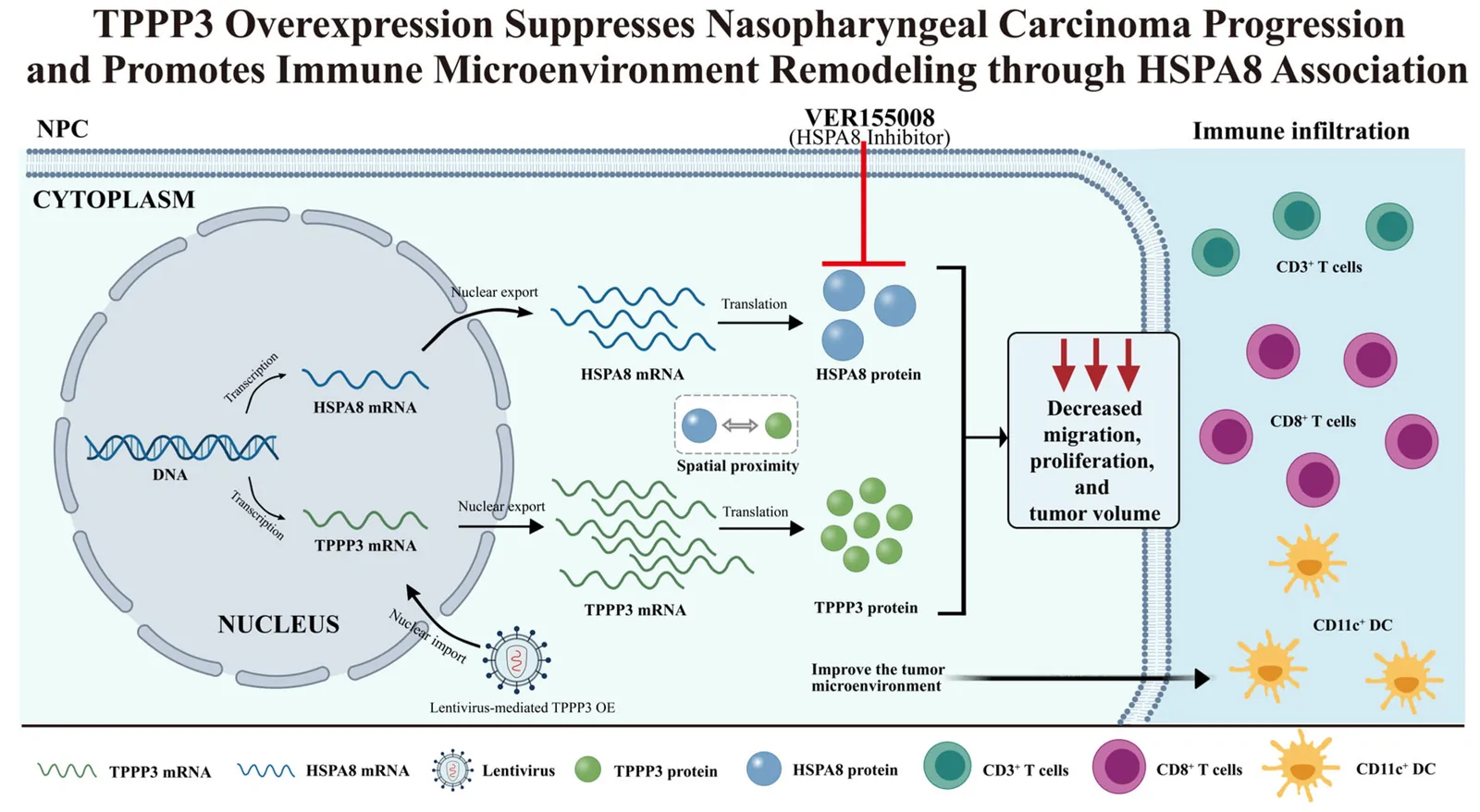
Schematic model of the TPPP3-HSPA8 functional association and immune modulation in nasopharyngeal carcinoma. TPPP3 overexpression suppresses malignant phenotypes and promotes T-cell/DC infiltration; its interaction with HSPA8 is functionally relevant, as HSPA8 inhibition by VER155008 synergizes to potentiate these antitumor effects.

This study further expanded its mechanism by linking the known anti-tumor function of TPPP3 in NPC with its immune cell composition in the tumor microenvironment and HSPA8-mediated protein homeostasis (Figure 7). Single-cell transcriptome data showed that the transcript of TPPP3 was almost undetectable in most HNSCC and NPC cell populations, but differential analysis showed that it was more enriched in NPC epithelial cells. This low absolute abundance and high relative specificity pattern suggests that TPPP3 has an intrinsic role in tumor cells and helps to explain the relationship between its transcription profile and the phenotypic changes observed after overexpression. Inhibition of xenograft growth was observed in both HK-1 and C666-1 models, which is consistent with previous studies that TPPP3 can limit the growth of NPC and further support its tumor suppressor activity in nasopharyngeal carcinoma.

The immune findings extend this tumor-intrinsic effect to the composition of the NPC microenvironment. TPPP3 overexpression increased intratumoral CD3^+^ T cells, CD8^+^ T cells, and CD11c^+^ dendritic cells, a coordinated pattern that is compatible with improved recruitment or retention of antigen-presenting and effector immune populations. This observation aligns with previous bioinformatic associations between TPPP3 expression and immune-related pathways in head and neck cancer. In the Transwell system, a modest increase in the CD3^+^CD8^+^ T-cell proportion was observed in some donors, whereas the pooled effect did not reach statistical significance. PBMC composition and responsiveness vary substantially among individuals, and the indirect co-culture system does not reproduce dendritic-cell function, stromal signaling, vascular trafficking, extracellular matrix, or direct intercellular contact. The stronger in vivo phenotype therefore supports a model in which TPPP3-associated immune remodeling emerges from multicellular interactions within the tumor microenvironment rather than from a single PBMC-directed soluble signal.

HSPA8 may serve as a molecular bridge to link TPPP3-dependent tumor inhibition with cellular protein homeostasis. In immunoprecipitation experiments, HSPA8 was continuously enriched in both NPC cell lines, and immunofluorescence showed that the two proteins had similar cytoplasmic distribution. Given the extensive involvement of HSPA8 in protein homeostasis and antigen processing, its association with TPPP3 suggests that this relationship may affect both tumor cell maintenance and immune recognition. The slight decrease in TPPP3 protein level after VER155008 treatment further indicated that the function of HSPA8/HSP70 helper protein may affect the stability of TPPP3 or its surrounding protein network. Taken together, these observations support a functional link between TPPP3 and HSPA8, although the detailed molecular structure of the association and its downstream effectors need to be further elucidated. Additionally, given the established role of HSPA8 in MHC-II stabilization and antigen presentation [21], future studies should investigate whether the TPPP3-HSPA8 association modulates MHC-I or MHC-II expression on NPC cells, which could represent a mechanism by which TPPP3 influences T-cell recognition and immune infiltration. While VER155008 is a well-established HSP70 inhibitor, off-target effects cannot be excluded. Genetic inhibition of HSPA8 via siRNA knockdown or CRISPR/Cas9 knockout will be required to confirm that the observed phenotypes are specifically attributable to HSPA8 inhibition, and this represents a key direction for future validation.

Pharmacological perturbation of the HSPA8/HSP70 chaperone network strengthened the antitumor phenotypes associated with TPPP3 overexpression. VER155008 further reduced cell migration and clonogenic growth, shifted cell-cycle distribution, suppressed xenograft progression, reduced Ki-67 staining, and increased CD3^+^ T-cell infiltration. In the wound healing assay performed under serum-free conditions, the reduced closure in TPPP3-overexpressing cells likely results from a combination of migration and proliferation. These concordant in vitro and in vivo effects indicate that TPPP3-overexpressing NPC cells retain a dependence on HSPA8/HSP70-mediated proteostasis and that disruption of this network can amplify tumor growth restraint and immune remodeling. The findings place proteostatic regulation alongside cytoskeletal control as a potential determinant of TPPP3 function in NPC and suggest that the TPPP3-HSPA8 association may represent a biologically relevant vulnerability in tumors with preserved or enhanced TPPP3 activity.

Beyond T-cell infiltration, several questions remain for future investigation. First, whether TPPP3 overexpression modulates immune checkpoint molecules—including PD-L1, CTLA-4, TIGIT, or LAG3—on NPC cells or infiltrating immune cells is unknown; addressing this could inform the rational combination of TPPP3-targeting strategies with immune checkpoint blockade. Second, while conventional IHC provided quantitative assessment of individual immune populations, multiplex immunofluorescence or spatial transcriptomic profiling would be required to fully characterize the spatial organization and functional interactions of immune cells within the TPPP3-overexpressing tumor microenvironment. Third, expanding immune profiling to include dendritic-cell maturation markers (CD80, CD86, HLA-DR), macrophage polarization markers (CD86, CD206), and NK-cell populations would provide a more complete picture of TPPP3-mediated immune modulation. Specifically, future studies should evaluate T-cell activation (CD69, CD25), proliferation (Ki-67), cytotoxicity (granzyme B, perforin), and cytokine production (IFN-γ, TNF-α) to determine whether the increased infiltration translates into effective anti-tumor immunity.

It should be pointed out that there are some limitations. Although HSPA8 has been identified as a TPPP3-related protein by IP-MS, its binding domain and precise molecular interface have not been clearly mapped. Donor heterogeneity may introduce variability in PBMC co-culture experiments, although the in vivo results of the two cell lines are consistent. Subsequent studies will focus on identifying the secretory factors regulated by TPPP3 that mediate immune cell recruitment and verifying its functional dependence on HSPA8 through genes. At the same time, optimizing the inhibitory effect of HSPA8 and testing its compatibility with chemotherapy and radiotherapy or immune checkpoint blockade combination therapy will be an important next step to promote its clinical application. Furthermore, validation in independent clinical cohorts is essential to establish the translational relevance of our findings. Future studies should evaluate TPPP3 and HSPA8 protein expression in NPC tissue microarrays and correlate these with immune infiltration patterns, clinical parameters, and patient outcomes. This would provide the necessary clinical evidence to support the therapeutic potential of targeting the TPPP3–HSPA8 association in NPC. Finally, the study relies predominantly on overexpression and pharmacological inhibition without complementary loss-of-function or rescue experiments to establish causality. These limitations will be addressed in our ongoing and future studies.

In summary, our findings suggest that TPPP3 and HSPA8 are functionally associated in modulating NPC progression through protein homeostasis. Interference with HSPA8 activity enhanced the anti-tumor and immunomodulatory effects conferred by TPPP3, pointing to a potential combination strategy for NPC treatment.

## 5. Conclusions

Collectively, this study establishes TPPP3 as a tumor-suppressive determinant in NPC and extends its biological role to remodeling of the local immune microenvironment. TPPP3 overexpression limited xenograft growth and increased intratumoral T-cell and dendritic-cell infiltration, while HSPA8 was reproducibly identified as a TPPP3-associated protein across two NPC models. Pharmacological disruption of the HSPA8/HSP70 chaperone network further intensified anti-migratory, anti-clonogenic, cell-cycle, and in vivo tumor-suppressive effects and enhanced CD3^+^ T-cell infiltration. These convergent findings suggest a functional association between TPPP3 and HSPA8 that integrates tumor-cell proteostasis with immune regulation and nominate HSPA8-dependent chaperone activity as a candidate therapeutic vulnerability for further evaluation in NPC.

## Data Availability

The data presented in this study are available on request from the corresponding author.

## Supplementary Materials

The following supporting information can be downloaded at: https://www.mdpi.com/article/10.3390/cancers18172851/s1, Figure S1. Dose-response curves of VER155008 in HK-1 and C666-1 cells. Cells were treated with increasing concentrations of VER155008 (0, 2, 4, 6, 8, 10 μM) for 48 h, and cell viability was assessed by CCK-8 assay. Data are presented as mean ± SD from three independent experiments. Based on these results, 5 μM was selected as the working concentration for subsequent in vitro functional assays, as it effectively inhibited cell viability without causing excessive cytotoxicity, allowing sufficient signal detection for phenotype assessment. Table S1. Protein identification table. Table S2. Identification statistics. Table S3. Peptide identification table.
