# Supplementary material for: TPPP3 Overexpression Suppresses Nasopharyngeal Carcinoma Progression and Promotes Immune Microenvironment Remodeling Through HSPA8 Association
Source: Cancers (Basel). 2026 Sep 3;18(17):2851. doi: 10.3390/cancers18172851 (PMC13565496; doi:10.3390/cancers18172851)
Supplement: Supplementary file 1 [file cancers-18-02851-s001.zip › cancers-4494668 WB.pdf]

For Western blotting, we routinely cut the membrane based on molecular weight markers when target proteins differ sufficiently in size, enabling simultaneous probing of multiple targets on the same membrane. When molecular weights were too close for reliable cutting, we performed parallel runs using separate membranes loaded with the same amounts of the same lysates, run and transferred in parallel, and probed side by side using identical antibody solutions and exposure settings. The results from parallel membranes were strictly compared only under these controlled conditions, and representative blots were chosen based on consistency across at least three independent repeats.

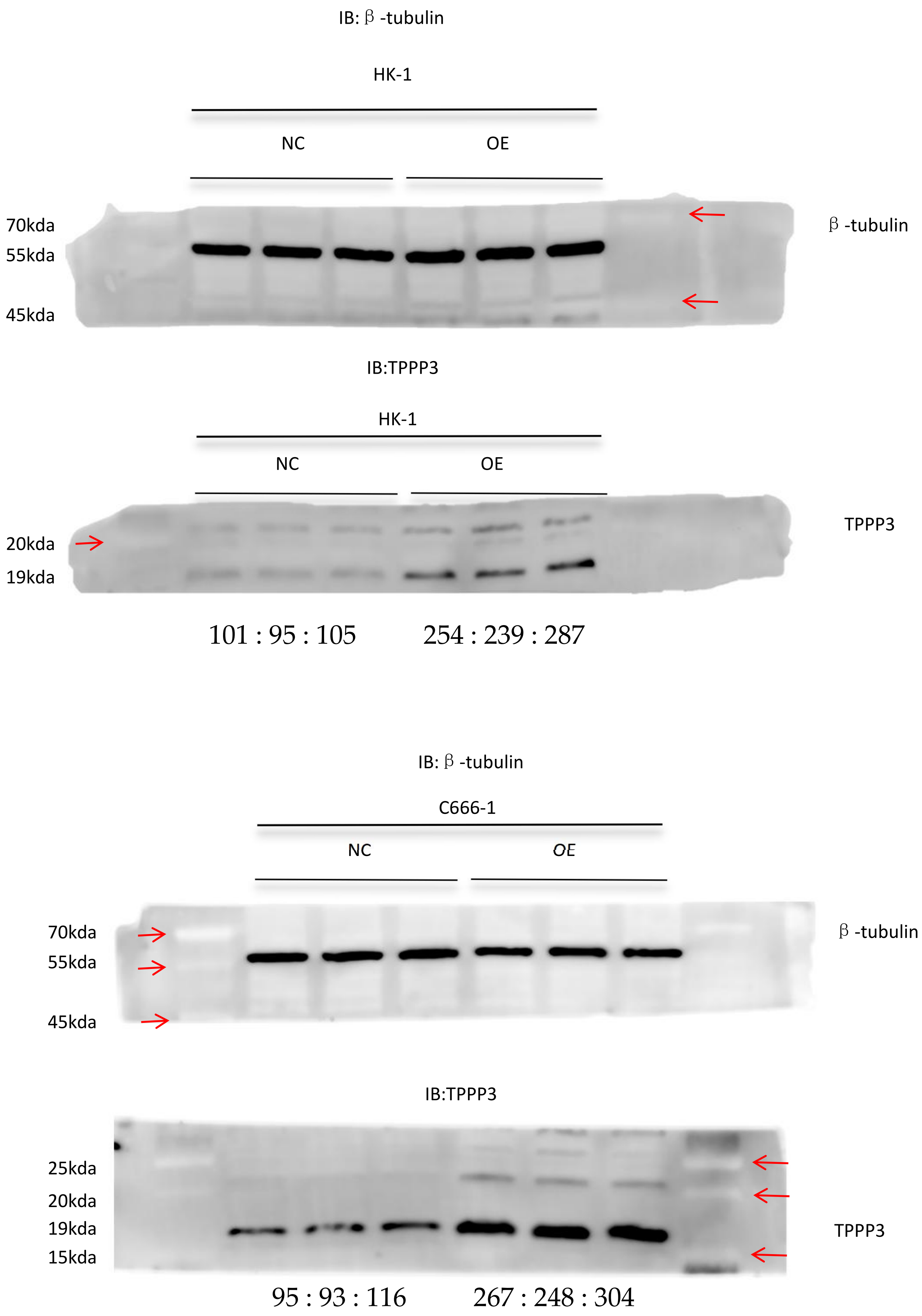

The red arrow indicates the marker.

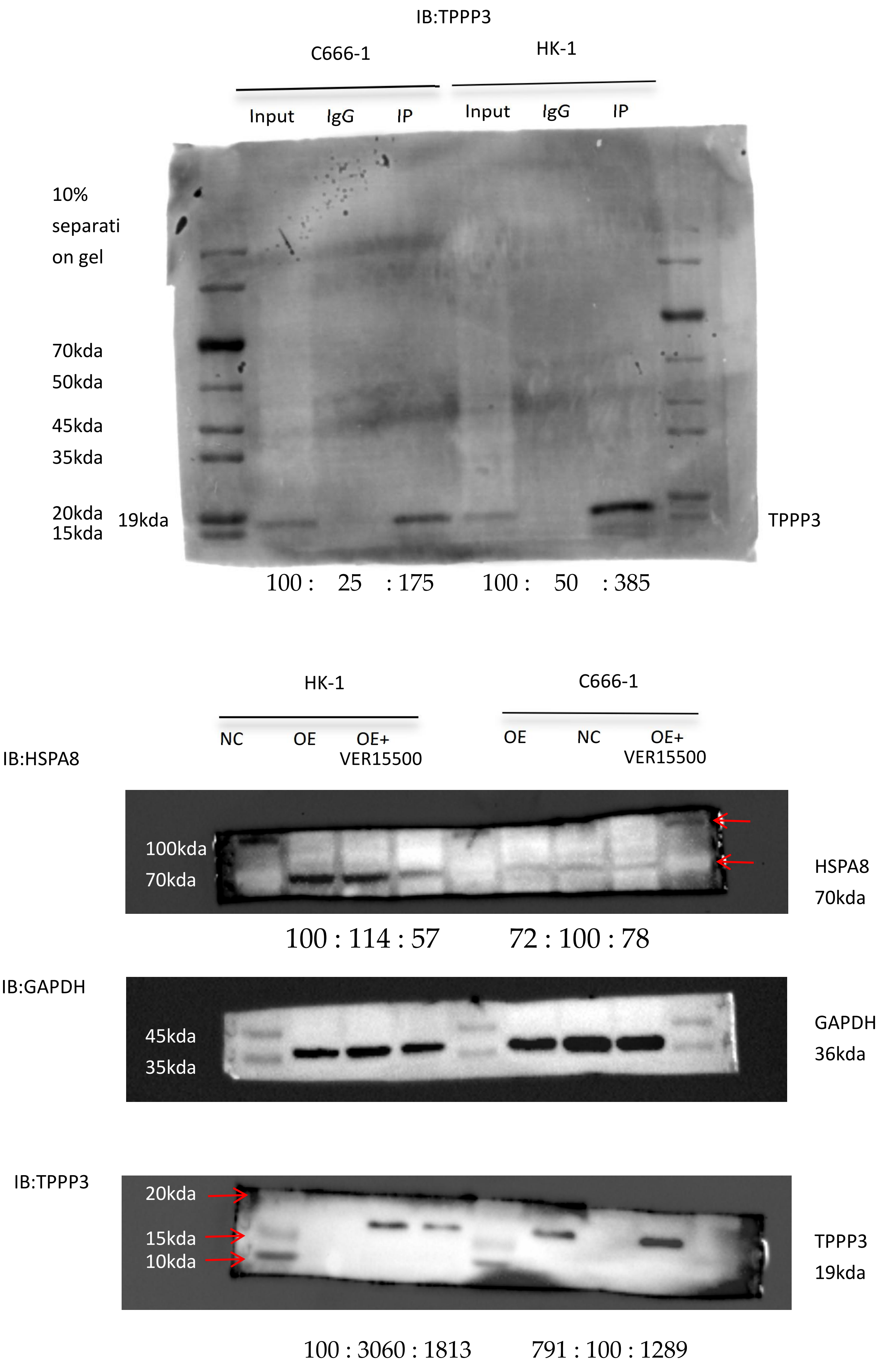
IB:TPPP3
C666-1
HK-1
Input
IgG
IP
Input
IgG
IP
10%
separati
on gel
70kda
50kda
45kda
35kda
20kda
15kda
19kda
TPPP3
100 : 25 : 175
100 : 50 : 385
HK-1
C666-1
NC
OE
OE+
VER15500
OE
NC
OE+
VER15500
IB:HSPA8
100kda
70kda
HSPA8
70kda
100 : 114 : 57
72 : 100 : 78
IB:GAPDH
45kda
35kda
GAPDH
36kda
IB:TPPP3
20kda
15kda
10kda
TPPP3
19kda
100 : 3060 : 1813
791 : 100 : 1289
