# Supplementary material for: TPPP3 Overexpression Suppresses Nasopharyngeal Carcinoma Progression and Promotes Immune Microenvironment Remodeling Through HSPA8 Association
Source: Cancers (Basel). 2026 Sep 3;18(17):2851. doi: 10.3390/cancers18172851 (PMC13565496; doi:10.3390/cancers18172851)
Supplement: Supplementary file 1 [file cancers-18-02851-s001.zip › Supplementary Figure S1.pdf]

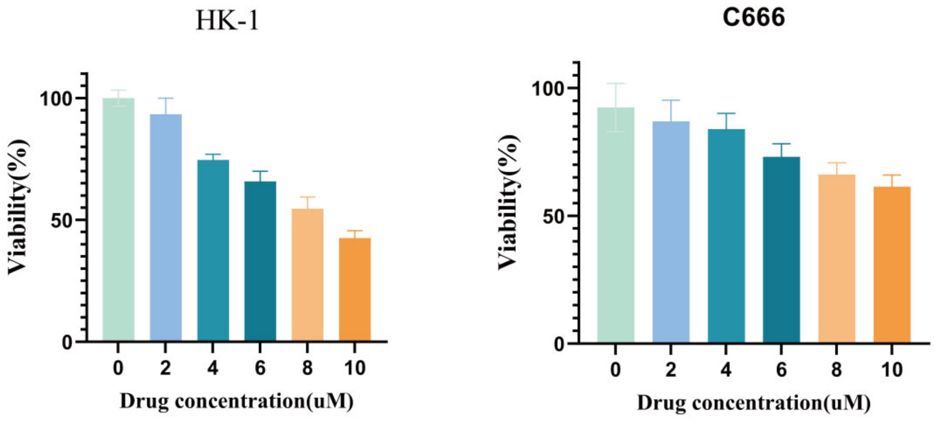

Supplementary Figure S1. Dose-response curves of VER155008 in HK-1 and C666-1 cells. Cells were treated with increasing concentrations of VER155008 (0, 2, 4, 6, 8, 10 μM) for 48 h, and cell viability was assessed by CCK-8 assay. Data are presented as mean ± SD from three independent experiments. Based on these results, 5 μM was selected as the working concentration for subsequent in vitro functional assays, as it effectively inhibited cell viability without causing excessive cytotoxicity, allowing sufficient signal detection for phenotype assessment.
